# Assessment of Acetylcholinesterase Activity Using Indoxylacetate and Comparison with the Standard Ellman’s Method

**DOI:** 10.3390/ijms12042631

**Published:** 2011-04-18

**Authors:** Miroslav Pohanka, Martina Hrabinova, Kamil Kuca, Jean-Pierre Simonato

**Affiliations:** 1 Faculty of Military Health Sciences, University of Defense, Trebesska 1575, 50001 Hradec Kralove, Czech Republic; 2 CEA-Grenoble, LITEN/DTNM/LCRE, 17 rue des Martyrs, 38000 Grenoble, France; E-Mail: jean-pierre.simonato@cea.fr

**Keywords:** acetylcholinesterase, indoxylacetate, 5,5′-dithio-bis-2-nitrobenzoic acid, Alzheimer’s disease, oxime reactivator, enzyme activity, nerve agents

## Abstract

Assay of acetylcholinesterase (AChE) activity plays an important role in diagnostic, detection of pesticides and nerve agents, *in vitro* characterization of toxins and drugs including potential treatments for Alzheimer’s disease. These experiments were done in order to determine whether indoxylacetate could be an adequate chromogenic reactant for AChE assay evaluation. Moreover, the results were compared to the standard Ellman’s method. We calculated Michaelis constant Km (2.06 × 10^−4^ mol/L for acetylthiocholine and 3.21 × 10^−3^ mol/L for indoxylacetate) maximum reaction velocity V_max_ (4.97 × 10^−7^ kat for acetylcholine and 7.71 × 10^−8^ kat for indoxylacetate) for electric eel AChE. In a second part, inhibition values were plotted for paraoxon, and reactivation efficacy was measured for some standard oxime reactivators: obidoxime, pralidoxime (2-PAM) and HI-6. Though indoxylacetate is split with lower turnover rate, this compound appears as a very attractive reactant since it does not show any chemical reactivity with oxime antidots and thiol used for the Ellman’s method. Thus it can be advantageously used for accurate measurement of AChE activity. Suitability of assay for butyrylcholinesterase activity assessment is also discussed.

## 1. Introduction

Acetylcholinesterase (AChE; EC 3.1.1.7.) is an enzyme participating in cholinergic neurotransmission. It breaks down acetylcholine which terminates the neurotransmission process [1]. AChE activity is inhibited by many compounds. The number of known inhibitors is rather extensive. Two main types of inhibitors can be distinguished from a practical point of view: toxins and drugs [2]. From a mechanistic point of view, the inhibitors are compounds with different structural motives as they can bind to the esteratic part of the active site by esterification of serine hydroxyl, or interact with the alpha anionic part of the active site, the aromatic gorge and the peripheral anionic site [3].

Assay of AChE activity can serve for diagnosis after potential exposure to organophosphorus or carbamate pesticides and nerve agents [4,5]. It can also be used for verification of treatment effectiveness, e.g., for Alzheimer’s disease therapy [6]. Novel drugs for Alzheimer’s disease or antidotal therapy are tested by *in vitro* methods when AChE is implicated in the treatment process [7,8]. Assay of nerve agents and selected pesticides by devices with AChE is another application of this enzyme [9]. Experimental protocols for AChE activity assay have been proposed. Unfortunately, the mechanism of AChE activity assay had limitations that can preclude its use in some pharmacological or toxicological experiments. The most common assay is based on Ellman’s method using an alternative substrate acetylthiocholine and 5,5’-dithio-bis-2-nitrobenzoic acid (DTNB). The reaction results in production of 5-thio-2-nitrobenzoate that has yellow color due to the shift of electrons to the sulfur atom. The method was developed by Ellman and coworkers in the early 1960s [10] and it is still used up to now, generally with significant modifications [11]. The Ellman’s method is particularly limited for testing antidots against organophosphorus AChE inhibitors or for measuring AChE activity in samples of such treated individuals. The antidots contain reactive oxime group splitting DTNB and provide false positive reaction in a process called oximolysis [12]. In this work we present experiments to determine AChE activity assay using indoxylacetate as an alternative substrate. We introduce a new alternative protocol to the Ellman’s method, which could be of high interest when DTNB may generate unwanted side reactions.

## 2. Results

**Figure 1.**
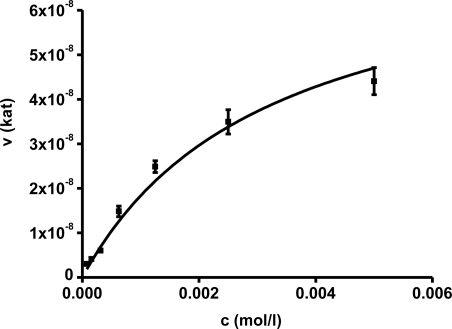
Saturation curve for acetylcholinesterase (AChE) and indoxylacetate as a substrate. The plot was fitted by Hill equation. Error bars indicate standard deviation for n = 4.

**Figure 2.**
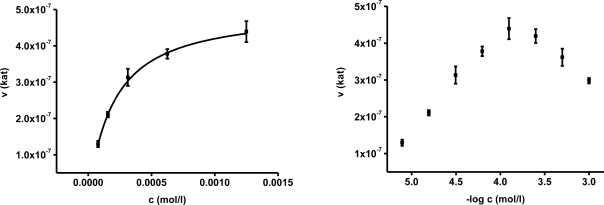
Saturation curve for AChE and acetylthiocholine chloride as a substrate. Semilagirmic is presented on the right. Plot on the left was fitted by the Hill equation. Error bars indicate standard deviation for n = 4.

In the first part of experiments, activity of AChE was assessed for different concentrations of substrates. The concentrations ranged from 10^−2^ to 10^−7^ mol/L and the assays were repeated four times. Saturations curves were constructed for the calculated enzyme activities. They are depicted in Figures 1 and 2. Acetylthiocholine above a concentration of 10^−4^ mol/L inhibited AChE activity. For toxicological and pharmacological testing, concentration of substrate of 10^−4^ mol/L was chosen as optimal. Indoxylacetate did not inhibit AChE up to the highest tested concentration, *i.e*., 5 × 10^−3^ mol/L. However, it has to be mentioned that indoxylacetate and indigo are not highly soluble compounds. Octanol water partition coefficient K_OW_ was calculated at 1.65 for indoxylacetate and 3.11 for indigo using EPI Suit software. The same software was used in order to estimate water solubility, 943 mg/L (5.39 mmol/L) for indoxylacetate and 109 mg/L (0.42 mmol/L) for indigo.

Biochemical parameters were calculated in the second step using non-linear regression. The best fitting was found for no cooperativity model (n = 1). The calculated K_m_ and V_max_ are presented in Table 1. K_m_ for acetylthiocholine and indoxylacetate were (2.06 ± 0.35) × 10^−4^ and (3.21 ± 0.31) × 10^−3^ mol/L, respectively. The V_max_ value was equal to (4.97 ± 0.42) × 10^−7^ kat for acetylthiocholine and (7.71 ± 0.56) × 10^−8^ kat for indoxylacetate.

We also investigated potency of butyrycholinesterase (BuChE) to split indoxylacetate and substitute AChE in this way. We prepared a standard solution of BuChE and AChE with activity 5 × 10^−7^ kat in 25 μL. The activity of BuChE was assessed using butyrylthiocholine as a substrate and the protocol described in Section 4.2 for substrate level 1 mM. Activity of AChE and BuChE using 1 mM indoxylacetate were (2.96 ± 0.53) × 10^−8^ and (7.86 ± 0.65) × 10^−8^ kat, respectively. Ratio of affinity AChE/BuChE toward indoxylacetate was calculated to be 2.65.

**Figure 3.**
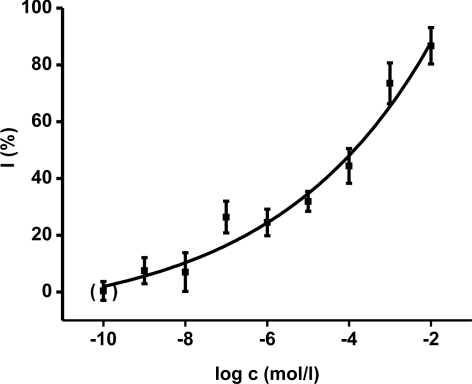
Calibration for ethyl paraoxon (logarithm of molar level) using AChE based assay with indoxylacetate as substrate. The absorbance shift is recalculated to percent of inhibition (I). The point in brackets was achieved by application of phosphate buffered saline instead of paraoxon. The error bars indicate standard deviation for four experiments.

**Figure 4.**
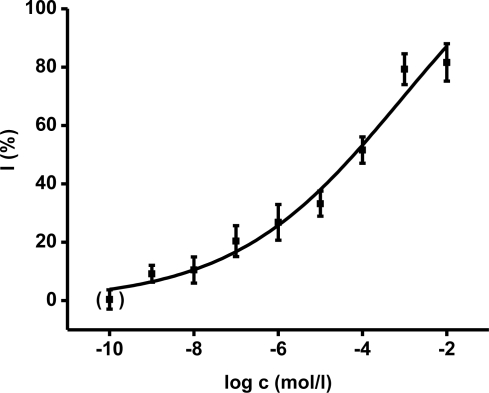
Calibration for ethyl paraoxon using AChE based assay with acetylthiocholine as substrate. The description is the same as for Figure 5.

Ethyl-paraoxon organophosphate was chosen as a model inhibitor. The final concentration of paraoxon in one cuvette ranged from 10^−9^ to 10^−2^ mol/L. The calibration curves (Figures 3, 4) were constructed for acetylthiocholine as well as indoxylacetate. The achieved enzyme activity and its decrease were re-calculated to percent of inhibition (I) due to better comparison of both methods. The limit of detection was calculated as signal to noise (control) ratio equal to three. The reached limit of detection for paraoxon was 10^−7^ mol/L for both methods.

2-PAM, obidoxime and HI-6 were tested as standard oxime reactivators. AChE was inhibited by ethyl-paraoxon up to 95% which means that 5% activity of AChE remained. The found return of activity was calculated as percent of reactivation *i.e.*, percent of activity from the original AChE activity before inhibition. Oxime reactivators were evaluated in the two final concentrations in cuvette at 10^−4^ and 10^−5^ mol/L. The calculated reactivation efficacies are summarized in Table 2. Comparing the reactivation efficacies, the achieved percent of reactivation were not significantly different on probability level 0.01 < P ≤ 0.05 or P ≤ 0.01 for both substrates.

The spontaneous interaction between indoxylacetate and oxime reactivators was examined with the negative result. There was no significant increase of absorbance due to oxime reactivator, whereas the Ellman’s method was quite sensitive to interference by oxime reactivators. The tested drugs provided false positive results because they spontaneously reacted with DTNB providing yellow colored 5-thio-2-nitro benzoic acid. The false positive reaction was approximately equal to the absorbance shift provided by 5 × 10^−7^ kat of AChE.

Potential interferences of biological matrices were tested using fresh rat blood. Blood was lysed using deionized water (blood:water—1:4). After spinning at 3,000 *× g*, absorbance at 670 nm was measured in supernatant. The found absorbance was >2.0. The measurement was repeated using supernatant diluted five-times (absorbance ∼0.50), ten-times (absorbance ∼0.25), twenty-times (absorbance ∼0.10), and forty-times (absorbance ∼0.05).

## 3. Discussion

Basic enzyme constants were calculated using both methods: Michaelis constant K_m_ and maximum reaction velocity V_max_. This experiment was mandatory in order to assess the functionality of the method. The present literature contains significant variations for the evaluation of AChE enzymatic parameters. The reported parameters differ notably due to different methods of AChE isolation, AChE origins, and physico-chemical conditions of storage, as mentioned e.g., by Barteri *et al.* for electric eel AChE [13]. The Michaelis constant measured in this work is approximately ten-times lower than the K_m_ reported for (*Rattus novergicus*) brain AChE: 2.65 mmol/L [14] but it is in a very good agreement with the one found for electric eel AChE: 0.13–0.15 mmol/L [15] for the substrate acetylthiocholine iodide. Higher K_m_ values are also known for common housefly (*Musca domestica*) AChE: 1.80 mmol/L [16]. Lower value than for rats and housefly are reported for human AChE: 0.98 [17]. The above mentioned Michaelis constants were achieved for acetylthiocholine as substrate. Unfortunately, the measured Michaelis constant using indoxylacetate cannot be compared with the literature as it is not available in the enzyme databases (Brenda) and papers. The fact that AChE is not inhibited in excess of indoxylacetate in contrast to acetylthiocholine is a novel very interesting output. Indoxylacetate was considered as a suitable chromogen and fluorogen in cholinesterase based assays [18,19]; however, the biochemical parameters have not yet been assessed. Tests aimed at comparison of AChE and BuChE sensitivity toward indoxylacetate as a substrate proved suitability of indoxylacetate as a universal substrate for cholinesterases. BuChE have even nearly three times higher affinity to indoxylacetate when compared to AChE. In regard to this fact, selective inhibitor of BuChE, tetraisopropyl pyrophosphoramide (iso-OMPA), shall be added into cuvette when only AChE is demanded to be assayed and BuChE interference could occur. Thus organized experiment will not be affected by BuChE due to iso-OMPA action and it is reliable for e.g., AChE assay in blood samples [20]. Biochemistry of BuChE was not a primary objective in this experiment; however, work devoted to the issue would be perspective in the future.

The comparison we performed with both indoxylacetate and acetylcholine as substrates shows similar calibration plots for paraoxon assay. The fact that evaluation of standard oxime reactivators shows the same reactivation potency is a very promising result. It raises the suitability of indoxylacetate as a new substrate for *in vitro* preliminary characterization of novel drugs. Moreover, contrary to the Ellman’s method reagents, the indoxylacetate does not directly react with oxime reactivators like DTNB [12,21]. In addition, indoxylacetate does not interact with thiols like DTNB with e.g., reduced glutathione [22].

However, it has to be mentioned that beside significant advantages, indoxylacetate has some limitations that should be taken in consideration. First, the calculated maximum reaction velocity is nearly ten-times lower for indoxylacetate. The lower turnover rate has to be compensated by prolonging assay time or increasing AChE amount in suspension. The poor solubility in water of both indoxylacetate and indigo must also be pointed out. Indoxylacetate and indigo have rather high octanol water partition coefficient.

The experimental data reported herein demonstrate that indoxylacetate can be recommended as a suitable substrate for toxicological or pharmacological characterization of new compounds implicated in AChE activity modulation. It could be a valuable alternative to the assay protocols based on Ellman’s method [23–25]. Pharmacological testing of anticholinersterase acting compounds is frequently based on blood AChE assay [13]. As seen in the results section, blood interference at 670 nm is quite low. Even five-times diluted blood lysate had low interference at 670 nm to be spectrophotometrically assayed. This is unlike the Ellman method where blood interference is a serious problem [5,13]. Suitability of indoxylacetate for blood cholinesterases assay can be inferred. Moreover, the assay is transmittable into 96-wells microplates allowing the lowering of costs per assay.

## 4. Experimental Section

### 4.1. Chemicals

AChE (electric eel, *Electrophorus electricus*, origin; 3.33–16.7 μkat/mg *i.e.*, μmol/s × mg), acetylthiocholine chloride, DTNB, ethyl-paraoxon, indoxylacetate and phosphate buffered saline (PBS) in tablets were purchased from Sigma-Aldrich. Organic solvents were of analytical grade. Sorbents and alkalines mixed with ethanol were used for paraoxon containing mixtures and disposable tools decontamination in a standard protocol. Oxime drugs including pralidoxime chloride (2-PAM; [(*E*)-(1-methylpyridin-2-ylidene)methyl]-oxoazanium chloride), obidoxime chloride (1,1’-[oxybis(methylene)]bis{4[(*E*)-(hydroxyimino)methyl]pyridinium}dichloride) and asoxime chloride (HI-6; [(*Z*)-[1-[(4-carbamoylpyridin-1-ium-1-yl)methoxymethyl]pyridin-2-ylidene]methyl]-oxoazanium dichloride) were previously synthesized at the Department of Toxicology, Faculty of Military Health Sciences, University of Defence, Hradec Kralove, Czech Republic. Purity was confirmed by thin layer chromatography.

### 4.2. Ellman’s Method for AChE

**Figure 5.**
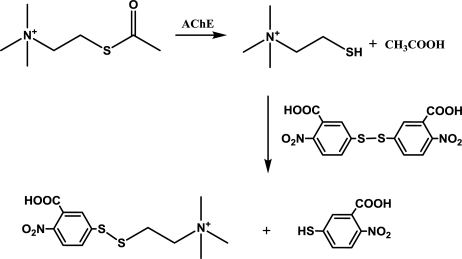
Chemical mechanism of Ellman’s method.

Ellman’s method was done with slight modifications of the reference [26,27]. The chemical principle is depicted in Figure 5. A disposable cuvette was consequently filled with 0.4 mL of 0.4 mg/mL DTNB, 25 μL of AChE solution (0.5 μkat in 1 mM acetylthiocholine), 425 μL of PBS, 50 μL of paraoxon in isopropanol or isopropanol alone. The reaction was started by adding 100 μL of acetylthiocholine chloride in a given concentration for assessment of K_m_ and V_max_ or 1 mM for toxicological and pharmacological investigations. Absorbance at 412 nm was measured immediately and after one minute. Enzyme activity was calculated estimating extinction coefficient ɛ = 14,150 M^−1^cm^−1^. The oxime drugs were tested in a similar protocol. 425 μL of PBS was reduced to 325 μL of PBS. Paraoxon was added in concentration providing 95% inhibition of AChE. Incubation time was set to 10 minutes. After that, 100 μL of oxime reactivator suspended in PBS was injected into the cuvette and kept for another 10 minutes. The reaction was started again by addition of acetylthiocholine.

### 4.3. AChE Activity Assay Using Indoxylacetate

**Figure 6.**
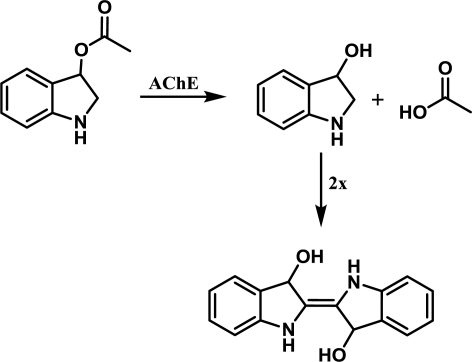
Chemical mechanism of indoxylacetate performance as AChE chromogenic substrate.

The reaction principle is depicted in Figure 6. The experiment was organized as a common spectrophotometric test using disposable cuvettes. One milliliter volume plastic cuvette was filled with 825 μL of PBS, 50 μL of paraoxon in isopropanol or isopropanol and 25 μL of AChE solution (the same activity as above) and gently shaken. The reaction was started by addition of indoxylacetate in 5% ethanol. This concentration was selected as a compromise between AChE inhibition and enabling of indoxylacetate solubility [9]. Absorbance of the mixture in the cuvette was measured at 670 nm shortly after mixture shaking and then after 30 minutes. Activity was calculated using extinction coefficient for which the value ɛ = 3,900 M^−1^cm^−1^ was adopted from literature [14]. Slight modification of the aforementioned protocol was made for oxime reactivators testing. 725 μL of PBS were placed into the cuvette instead of 825 μL. Paraoxon was injected in an amount providing 95% inhibition of AChE and the mixture was left for 10 minutes. Reactivation was triggered by addition of 100 μL of tested oxime reactivator and colorimetric reaction was started by addition of indoxylacetate 10 minutes after the reactivator. All experiments were performed under standard laboratory conditions (SATP).

### 4.4. Partition Coefficient and Water Solubility Calculation

EPI Suite (Office of Pollution Toxics and Syracuse Research Corporation; US Environmental Protection Agency) software was used in order to calculate octanol water partition coefficient and water solubility.

### 4.5. Statistics and Data Processing

Michaelis constant K_m_ and maximum reaction velocity V_max_ were calculated using non-linear regression and software Origin 8 SR2 (OriginLab Corporation, Northampton, MA, USA). The Hill equation was used throughout for variable cooperativity and non-cooperative model (n = 1). Origin 8 software was also used for data processing and inferential statistics. Significance of results was evaluated by one-way ANOVA with Tukey test. Both 0.01 < P ≤ 0.05 and P ≤ 0.01 probability levels were calculated.

## 5. Conclusions

Basic enzyme constants (Michaelis constant and maximum reaction velocity) were calculated for electric eel AChE and two substrates: acetylthiocholine and indoxylacetate. Performance of indoxylacetate in pharmacology and toxicology was evaluated, and the suitability to use indoxylacetate for cholinesterase based tests was demonstrated on ethylparaoxon as standard pesticide and 2-PAM, obidoxime and HI-6 as standard oxime reactivators drugs. We believe that this work could be useful in the development of alternative techniques to the Ellman’s method and that indoxylacetate could be of great interest in some new protocols.

## Figures and Tables

**Table 1.** Biochemical parameters of electric eel acetylcholinesterase (AChE) calculated using non-linear regression analysis.

***Substrate***	***Acetylthiocholine***	***Indoxylacetate***
***K_m_* (*mol/l*)**	(2.06 ± 0.35) × 10^−4^	(3.21 ± 0.31) × 10^−3^
***V_max_* (*kat*)**	(4.97 ± 0.42) × 10^−7^	(7.71 ± 0.56) × 10^−8^

K_m_—Michaelis constant; V_max_—maximum reaction velocity.

**Table 2.** Reactivation of ethyl-paraoxon inhibited AChE. The values indicate mean percent of reactivation for four experiments.

***Oxime*(*final concentration*)**	***Substrate—indoxylacetate***	***Substrate—acetylthiocholine***
***Obidoxime 10^−4^ mol/L***	46.1 ± 3.1	50.5 ± 2.5
***Obidoxime 10^−5^ mol/L***	26.6 ± 2.1	22.8 ± 1.7
***HI-6 10^−4^ mol/L***	7.4 ± 0.7	6.8 ± 1.0
***HI-6 10^−5^ mol/L***	7.0 ± 0.4	7.1 ± 1.2
***2-PAM 10^−4^ mol/L***	34.0 ± 1.7	31.2 ± 1.4
***2-PAM 10^−5^ mol/L***	11.0 ± 0.5	11.7 ± 1.3

2-PAM—pralidoxime; HI-6—asoxime.
